# Prehospital telemedicine support for urban stroke care: Analysis of current state of care and conceptualization

**DOI:** 10.1186/s12873-024-01142-3

**Published:** 2024-11-27

**Authors:** Daniel Weiss, Christian Rubbert, Marius Kaschner, Gregory Gordon Greiner, Nadja Kairies-Schwarz, Markus Vomhof, Andrea Icks, Linea Weitz, Hanna Hollenberg, Robin Jansen, Til Menge, Rüdiger J. Seitz, Sebastian Jander, Michael Bernhard, John-Ih Lee, Tobias Ruck, Sven Guenther Meuth, Bernd Turowski, Julian Caspers, Michael Gliem

**Affiliations:** 1 Department of Diagnostic and Interventional Radiology, Medical Faculty and University Hospital Düsseldorf, Heinrich-Heine-University Düsseldorf, Moorenstraße 5, 40225 Düsseldorf, Germany; 2 Institute for Health Services Research and HEs, Centre for Health and Society, Medical Faculty and University Hospital Düsseldorf, Heinrich-Heine-University Düsseldorf, Moorenstraße 5, 40225 Düsseldorf, Germany; 3 Department of Neurology, Medical Faculty and University Hospital Düsseldorf, Heinrich-Heine-University Düsseldorf, Moorenstraße 5, 40225 Düsseldorf, Germany; 4 Department of Neurology, Centre of Neurology and Neuropsychiatry, LVR-Klinikum, Heinrich-Heine-University Düsseldorf, Medical Faculty, 40629 Düsseldorf, Germany; 5 Department of Neurology, Marienhospital, Rochusstraße 2, Düsseldorf, 40479 Germany; 6 German Diabetes Center, Institute for Health Services Research and HEs, Leibniz Center for Diabetes Research at Heinrich-Heine-University Düsseldorf, Düsseldorf, Germany; 7 Emergency Department, Medical Faculty and University Hospital Düsseldorf, Heinrich-Heine-University Düsseldorf, Moorenstraße 5, 40225 Düsseldorf, Germany

**Keywords:** Stroke, Models of care, Outcome, Cost efficiency

## Abstract

**Background:**

The reduction of processing times in the treatment of acute ischemic stroke is of outstanding importance. Our objective is to analyze the acute stroke care chain from onset to treatment in a city in Germany comprising three stroke units. Additionally, we discuss solutions for detected treatment delays.

**Methods:**

We conducted an in-depth analysis of acute stroke care processing times across three local stroke centers in Düsseldorf among all emergency services transportations for suspected stroke. Isochrone mapping was performed to identify areas with prolonged transportation times.

**Results:**

Among the 1,714 transportations, 943 patients had confirmed strokes. Prehospital care constituted 58% of total emergency care time until imaging. Patients with confirmed stroke had reduced in-hospital times while patients receiving treatment experienced faster in-hospital times. Isochrone mapping revealed disparities in transportation times within the city.

**Conclusions:**

In conclusion, we identified confirmation of stroke symptoms as pre- and in-hospital and treatment eligibility as in-hospital process accelerators in stroke care. We propose the introduction of an in-ambulance video consulting model to accelerate contact to stroke-experts and accelerate processing times for patients eligible for treatment. Furthermore, we discuss the combination of in-ambulance video consulting with imaging and starting treatment outside traditional stroke centers, followed by transportation to a stroke center during thrombolysis, which might further accelerate treatment in specific cases.

**Supplementary Information:**

The online version contains supplementary material available at 10.1186/s12873-024-01142-3.

## Background

Acute ischemic stroke (AIS) is the leading cause of persistent disability and death in Western countries. Intravenous thrombolysis (IVT) and endovascular treatment (ET) are now established and efficient treatments [1–4]. However, minimizing the time between symptom onset and vessel recanalization is key to successful treatment of stroke patients, making rapid prehospital and in-hospital processes essential.

Many structural efforts have improved stroke care like prenotification by emergency medical services (EMS), guidance of patients to available stroke unit (SU) beds and training of specialized SU teams. Despite those improvements, only 10% of all stroke patients are treated and 1% of them are treated within the “golden hour” [5, 6].

Telemedicine has emerged as a valuable tool for prehospital stroke care, consistently reducing process times. Mobile Stroke Units (MSU) have shown promising results in reducing care times and improving patient outcomes, though cost constraints limit their widespread use [7].

With the current study, we endeavor to thoroughly examine the existing state of stroke care, particularly focusing on the time it takes to complete the pre-hospital and in-hospital stages of the rescue chain for all stroke patients admitted to one of the three Stroke Units (SUs) in a metropolitan region of Germany. Using the data gathered, we plan to model the cost difference between the existing model with the novel concept of using telemedicine during EMS transit to reduce delays in the time for patients to receive treatment.

## Methods

The assessment and analysis of processing times were conducted as part of a prospective observational study across three stroke centers (SC). The study was approved by the local ethics committee (ID: 2021–1494). Obligation for written informed patient consent was waived by the local ethics committee.

The city of Düsseldorf has approximately 650,000 inhabitants. There are three SCs in the city, including one comprehensive SC (CSC, *SC A*) and two primary SCs (PMC, *SCs B* and *C*). Endovascular treatment (ET) of stroke patients at *center 2* follows a drip-and-ship (DnS) principle, with eligible patients being transferred to the CSC 1. SC 3 operates on a drip-and-drive (DnD) basis, with an external neurointerventionalist arriving at the hospital.

### Processing times in pre- and in-hospital stroke care

An interdisciplinary healthcare proof system (IVENA) for coordination between EMS and hospitals has been implemented in Düsseldorf, which allows for digital advance notification of cases admitted by EMS. All EMS transportations with the IVENA patient admission codes “421 – Stroke / transient ischemic attack (TIA) / intracerebral hemorrhage (ICH) < 6 h “, “422 – Stroke / TIA / ICH 6–24 h “ and “425 – cerebral vessel occlusion for thrombectomy “ between July 2021 and June 2022 were prospectively included in each of the SCs. Data were collected from medical health records and documentation for quality assurance. Per standard operating procedure, transport of patient with AIS or TIA codes do not require emergency physician backing or contact.

The following time points were assessed: Onset-time, i.e., exact time when the first symptoms are observed; alarm-time, i.e., time of the emergency call for stroke suspicion to EMS; pickup-time, i.e., time when ambulance transfer, following on-scene care by EMS, is initiated; door-time, i.e., time when patient arrives at the SC; imaging-time, i.e., time when CT or magnetic resonance imaging (MRI) begins. For patients receiving IVT, needle-time was additionally assessed. All patients who received IVT were treated with Alteplase (rt-PA), none received tenecteplase. For patients undergoing ET, groin-puncture-time was assessed. For patients receiving ET with prior secondary transfer to the CSC, the arrival-time at the CSC was assessed.

### Isochrone mapping for identification of city parts with increased transportation times

Isochrone maps were generated using the web application of OpenRouteService (https://maps.openrouteservice.org/).

Initially, isochrones for 3, 6, 9, 12, and 15 min of transportation time were calculated for each SC and overlaid onto a single map. Subsequently, areas suitable for optimal allocation to the SCs were defined by assigning each area of the map to the SC with the shortest isochrone at the respective position.

Isochrones for five additional hospitals in the city without a stroke-unit were generated in 5-min intervals. For each area of the map, the time difference between transportation to the nearest SC and transportation to one of the other hospitals was calculated. An area was then assigned to one of the non-stroke-unit hospitals on the final map when time difference favored transportation to the non-stoke-unit hospital by at least 5 min.

### Health economic (HE) assessment of stroke care with and without prehospital telemedical care

The HE assessment was conducted from the hospital perspective to assess processing costs relevant for current stroke care. These costs encompass expenses related to stroke care, including the salaries of health care professionals, measured by involved professionals and the duration of processes.

We compared current stroke care with the novel concept of telemedicine for early patient-stroke expert contact during EMS transit. For the latter, we assumed that the transport time will not be extended by the use of in-transit telemedicine and that the door-to-imaging time for all patients will decrease to 10 min based on expert assumptions. In-transit telemedicine costs were calculated, anticipating a duration of 5 min and a 4:1 assistant physician to senior physician ratio.

Based on the different applicable collective agreements for the public service in 2022 (index year), the gross wages per minute for various professional groups involved in care were calculated. This calculation considered gross wages, working days, and vacation days to which they are contractually entitled. Due to potential variations in experience levels within groups such as paramedics, emergency paramedics, senior and assistant physicians, nursing staff, etc., as well as different applicable collective agreements, expert assumptions were utilized to obtain an average value for each group.

### Statistical analysis

Median and interquartile ranges of time intervals between the specified time points in the stroke rescue chain were calculated for all included patients with sufficient time data. Time intervals were tested for significant group differences using unpaired two-sided t-tests. *p*-values ≤ 0.05 were considered statistically significant.

General statistical analyses were conducted in SPSS Statistics 28 (IBM Corp., Armonk, NY, USA).

## Results

### Pre-hospital and in-hospital process times of emergency stroke care across the city

**Fig. 1:**
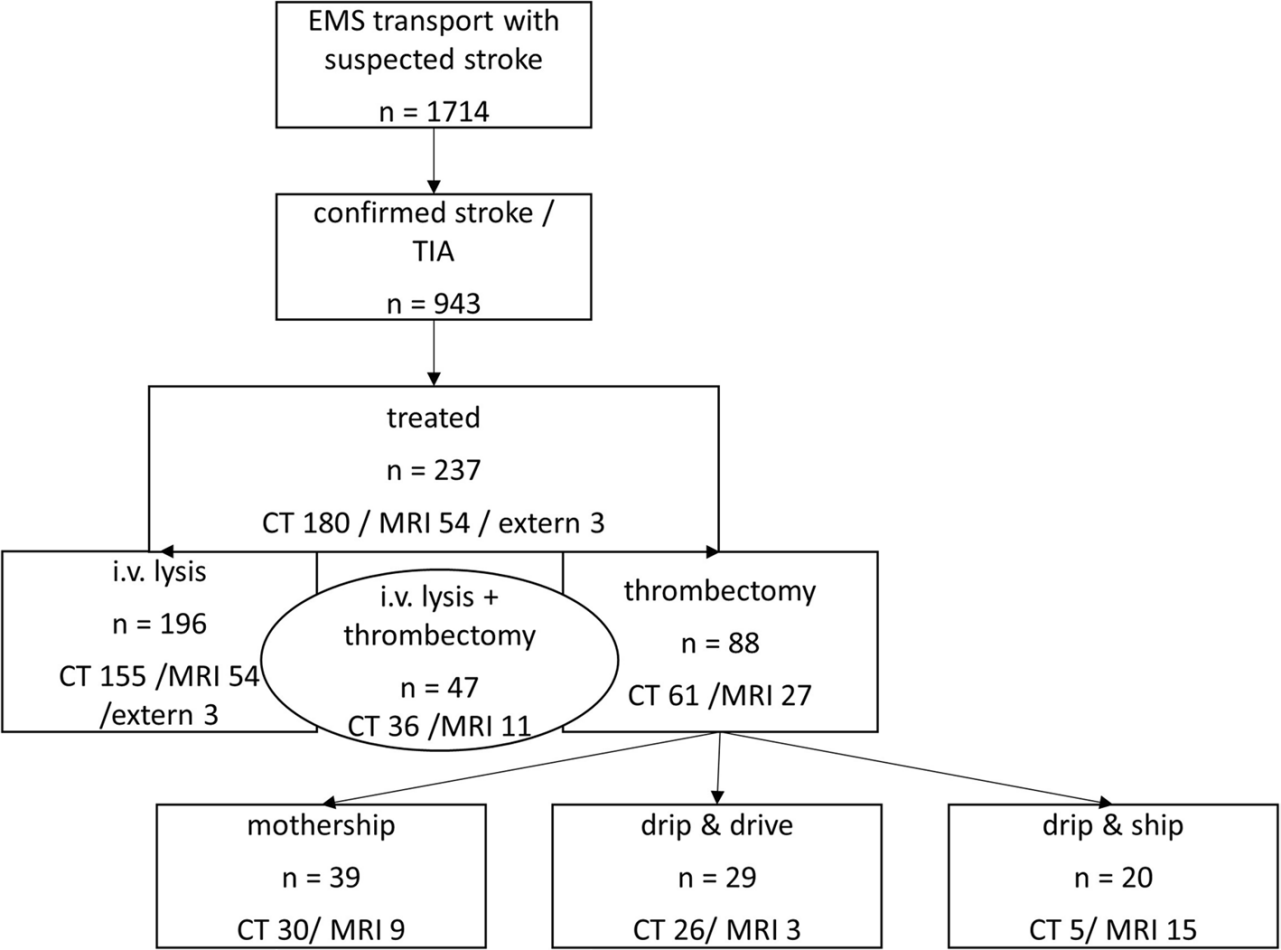
Flow-chart of case inclusion. EMS = emergency medical services; TIA = transient ischemic attack; CT = computed tomography; MRI = magnetic-resonance-tomography

During the one-year observation period (July 2021 – June 2022), a total of 1,714 transportations by local EMS with suspicion of stroke, as indicated by specified prenotification alert codes, were assessed. In 943 of these cases, AIS or a TIA was confirmed in the clinical evaluation at the respective SC’s emergency department. The diagnosis was made according to the recommendations of current guidelines and included a comprehensive diagnostic approach. In the case of a TIA, differentiation can sometimes be challenging when symptoms have fully resolved before examination by a neurologist, and it therefore relies on their expertise and the results of clinical and instrumental examinations [8]. Among these 1,714 initial transportations, 75 patients were found to have some form of cerebral hemorrhage, including intracerebral or subarachnoid hemorrhage, or a subdural hematoma. The remaining 696 patients had a different cause for their symptoms, consistent with a stroke mimic. A flow-chart of case acquisition is illustrated in Fig. 1 and related process times are depicted in Table 1..

**Table 1:** Process times for patients with incomplete datasets

Process time	Number of patients	Median (IQR) [minutes]	Mean (SD) [minutes]
Alarm-to-pickup	918	30 (24–37)	31.6 (± 11.1)
Pickup-to-door	909	14 (10–18)	14.7 (± 7.2)
Onset-to-door	1336	127 (62–378)	368.7 (± 1312.4)
Door-to-image	1628	32 (21–50)	43.9 (± 41.7)
Image-to-needle	185	17 (10–32)	22.5 (± 18.5)
Onset-to-needle	154	130 (90–215)	198.6 (± 243.9)
Image-to-groin (all)	85	79 (57.5–111.5)	83.9 (± 36.3)
Onset-to-groin (all)	86	195 (146–415)	313.8 (± 252.2)
Image-to-groin (MS)	39	57 (42–75)	60.3 (± 25.7)
Image-to-groin (DnS)	20	120.5 (106.75–128.75)	119.7 (± 20.3)
Image-to-groin (DnD)	26	82.5 (69.75–109.5)	91.9 (± 33.8)

*MS *Mothership principle, *DnS *Drip-and-ship principle, *DnD *Drip-and-drive principle

Of the 88 patients who underwent ET, 39 patients were directly admitted to a SC providing an on-site interventional neuroradiology for ET (“mothership” principle, MSP), 29 were admitted to a PMC where an external neurointerventionalist arrived for ET (“Drip and Drive” principle, DnD), and 20 patients were admitted to a PMC and secondarily transferred to the CSC for ET (“Drip and Ship” principle, DnS).

**Fig. 2:**
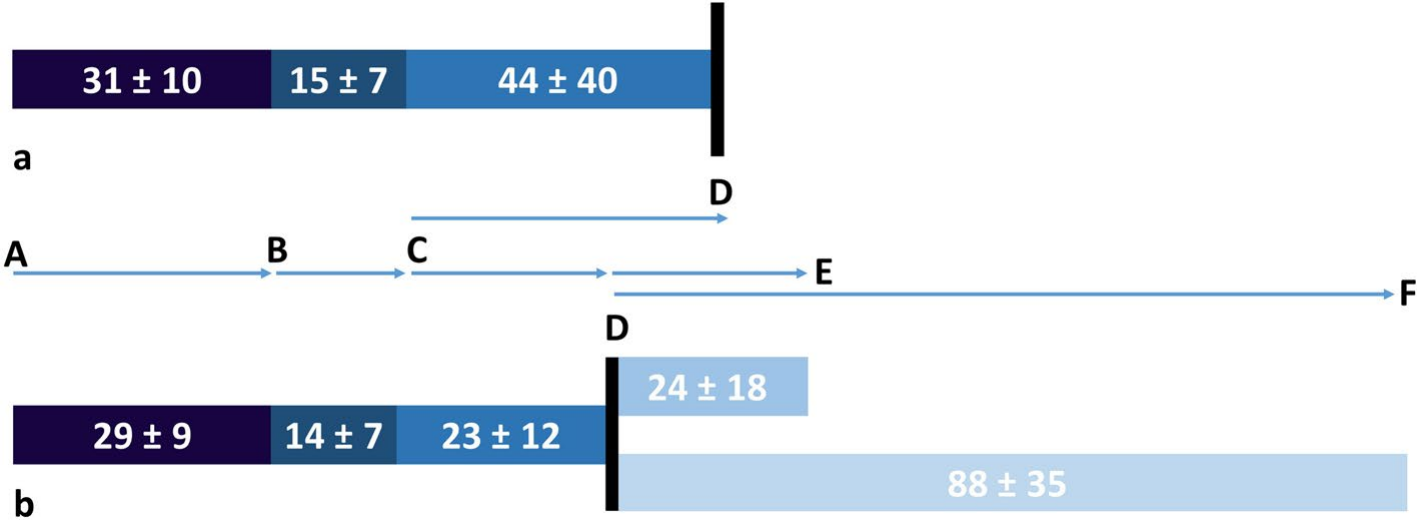
Timeline from alarm-to-imaging-time for a) all suspected stroke cases (*n* = 860) and b) treated patients (*n* = 121). **A** = alarm-time, **B** = pickup-time, **C** = door-time, **D** = imaging-time, **E** = needle-time, **F** = groin-puncture-time. The numbers in the bars correspond to the means and standard deviations of the time periods in minutes

Complete data on all time points, from the initial alarm to imaging, were available for 860 cases (Table 2, Fig. 2). For these, comparison of time intervals between confirmed strokes or TIAs (*n* = 519) and patients without stroke (*n* = 341) is given in Table 3.

**Table 2:** Process times for patients with complete datasets from alarm-to-imaging

Process time	Number of patients	median (IQR) [minutes]	Mean (SD) [minutes]
Alarm-to-pickup	860	29 (24–36)	30.9 (± 10.1)
Pickup-to-door	860	14 (10–18)	14.6(± 7.2)
Door-to-image	860	32 (22–50)	43.6(± 40.4)

**Table 3:** Process times for patients with complete datasets from alarm to imaging or onset-to-door: confirmed strokes/TIA vs. non-strokes

Process time	Number of patients		median (IQR) [minutes]		Mean (SD) [minutes]		*p*—value*
	stroke	non-stroke	stroke	non-stroke	stroke	non-stroke	
Alarm-to-pickup	519	341	28 (23–35)	31 (25–38)	29.8 (± 11)	32.6 (± 11)	< 0.001
Pickup-to-door	519	341	13(9–18)	14 (10–18)	14.4 (± 11)	14.9 (± 7)	0.296
Onset-to-door	455	274	127 (60–382)	123 (61–370)	330.6 (± 519)	304.0 (± 465)	0.474
Door-to-image	519	341	29 (21–43)	36 (25–57)	39.5 (± 11)	49.7 (± 46)	< 0.001

Complete time point data from the alarm to treatment were assessable for 121 out of the 236 treated patients (Table 4). These data points were compared with patients who did not receive any treatment (*n* = 398) in Table 4. The *p*-values in Tables 3 and 4 are based on a comparison of means using a parametric t-test, conducted after confirming normal distribution. Additionally, the door-to-image time for all treated patients who underwent MRI was 30.8 min (± 13.9), and for all treated patients who underwent CT, it was 21.3 min (± 14.0).

**Table 4:** Process times for patients with complete datasets from alarm to imaging or onset-to-door: treated vs. untreated

Process time	Number of patients		median (IQR) [minutes]		Mean (SD) [minutes]		*p*—value
	treatment	no treatment	treatment	no treatment	treatment	no treatment	
Alarm-to-pickup	121	398	28 (22–34)	29 (23–35)	28.7 (± 9)	30.2 (± 10)	0.135
Pickup-to-door	121	398	13 (10–18)	13 (9–18)	14.5 (± 7)	14.4 (± 8)	0.938
Onset-to-door	116	339	83 (54–173)	174 (69–483)	189.7 (± 296)	378.8 (± 568)	< 0.001
Door-to-image	121	398	21 (15–29)	32 (23–52)	23.4 (± 12)	44.4 (± 39)	< 0.001

Of all treated patients, 97 had complete time point data for the administration of IVT, and 40 patients who underwent ET had complete time points until groin puncture (Fig. 1, Table 5).

**Table 5:** Process times for patients with complete datasets from alarm to therapy

Process time	Number of patients	median (IQR) [minutes]	Mean (SD) [minutes]
Image-to-needle	97	18 (10–36)	23.6 (± 17.6)
Image-to-groin (all)	40	84.5 (64.5–116.25)	87.7 (± 35.4)
Image-to-groin (MS)	17	64 (40.5–88.5)	65.8 (± 30.6)
Image-to-groin (DnS)	12	124 (106.75–144.25)	124.0 (± 24.2)
Image-to-groin (DnD)	11	81 (69–89)	81.7 (± 17.2)

*MS* mothership principle, *DnS* drip-and-ship principle, *DnD* drip-and-drive principle

Of note, the total number of patients treated for AIS at the three SCs was much higher, as only cases directly admitted by EMS to one of the SCs based on specific IVENA codes for stroke suspicion were included for the study. Admissions with different transportation methods, IVENA codes, secondary admissions, or in-house strokes were not included.

There were significant time differences between patients with confirmed stroke and patients without stroke confirmation for alarm-to-pickup-time (*p* < 0.001), and for alarm-to-door-time (44.6 min vs 47.9 min, *p* < 0.001), but not for pickup-to-door-time (p = 0.296) with complete data sets (Table 3).

There were no significant time differences in prehospital times (alarm-to-pickup-time, alarm-to-door-time, pickup-to-door-time) between patients receiving treatment and untreated patients.

Furthermore, in-hospital care preceding imaging was significantly faster for patients with confirmed stroke compared to patients without stroke (39.5 min vs 49.7 min, *p* < 0.001) and between treated stroke patients and untreated stroke patients (23.4 vs. 44.4 min, *p* < 0.001) (Fig. 2, Tables 3 and 4).

**Fig. 3:**
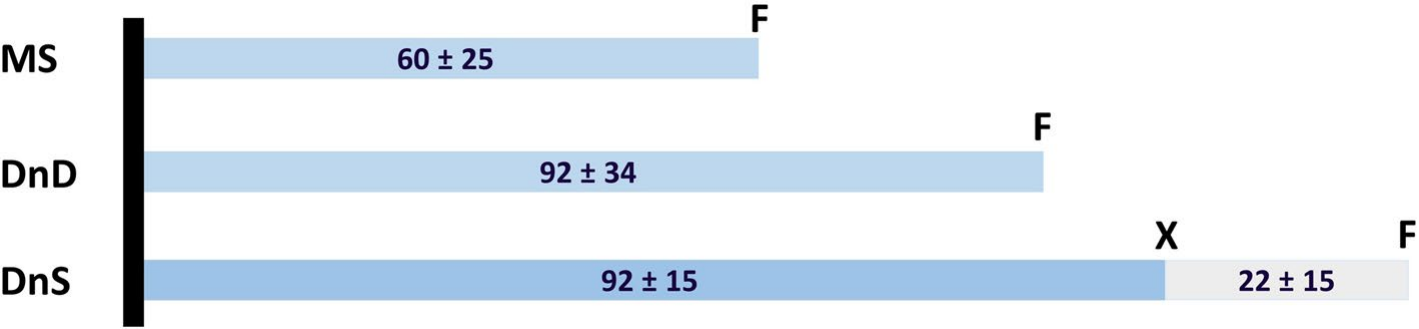
Therapy related times for patients who underwent endovascular treatment for patients with complete datasets from image to groin puncture. MS = mothership principle (*n* = 39), DnD = drip-and-drive principle (*n* = 26), DnS = drip-and-ship principle (*n* = 20), black bar = imaging-time, F = groin-puncture-time, X = arrival-time. The numbers in the bars correspond to the means and standard deviations of the time periods in minutes

In the group of treated patients, median imaging-to-needle-time was 21 min across all three centers, and median imaging-to-groin-puncture-time was 84.5 min across all three admission strategies (Table 4, Fig. 3).

Imaging-to-groin-puncture-times separated for each admission strategy are illustrated in Fig. 3. They were significantly lower for patients admitted with the MSP compared to DnD (*p* < 0.001) and DnS (*p* < 0.001). Furthermore imaging-to-groin-puncture-times for DnD were significantly lower compared to DnS (p = 0.001).

Regarding in-hospital workflows, we observed an average door-to-needle time of 46 min (± 24 min) with a median of 41 min (IQR: 27–61), and an average door-to-groin puncture time of 111 min (± 39 min) with a median of 108 min (IQR: 84–141).

The median mRS for all treated patients was 2 at discharge and 3 after three months.

### Isochrone mapping for analysis of transportation times

**Fig. 4:**
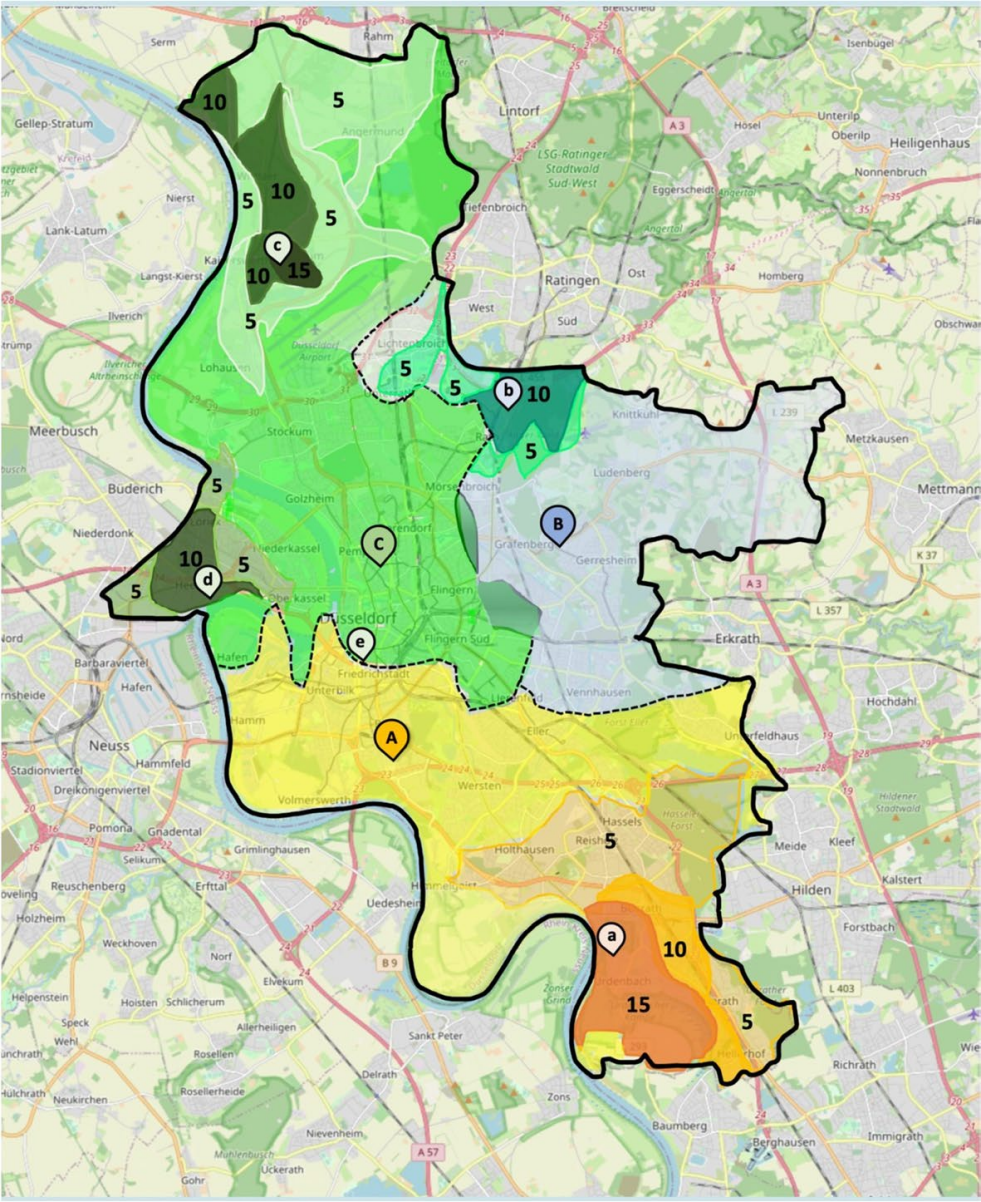
Isochrone maps for transport times for all stroke units. **A** comprehensive stroke center (yellow); **B** (blue), **C** (green) = primary stroke centers, a-d = general hospitals; numbers indicate the time benefit of transporting patients to the nearest CT (a-e) rather than the Stroke Unit in minutes

The mapping of isochrones for transportation times to the three SCs divided the city in three parts of comparable size (Fig. 4). It revealed substantial areas where transportation times exceeded 15 min to any SC. When additional isochrones for other hospitals were overlaid on the map possible time advantages for transportation to a non-SC hospital compared to transportation to the respective SC of up to 15 min became evident. Although hospital b is located within the area of shortest transportation time to SC B, it was allocated for transfer to SC C in the map as both hospitals belong to the same hospital provider group. However, the illustrated time advantages for transportation between hospital b and SC C also apply for the difference between hospital b and SC B.

### HE assessment

In the HE assessment, we found that suspected stroke cases incur average staff costs of €131.91. Patients who received therapy had lower costs due to shorter door-to-image times. The implementation of in-transit telemedicine could result in savings of €38.93 on average per patient, particularly in cases where stroke is not confirmed (€46.67 on average per patient). The use of in-transit telemedicine is cost-effective when door-to-image times are reduced to an average of 40.5 min. Time and staff cost savings are in line with findings of other cost-analyses across Europe [9–11]. Full results can be found in Supplement A.

## Discussion

The safety and effectiveness of stroke treatment hinge on time, and endeavors to expedite stroke care involve optimizing processes both before and after emergency department (ED) admission. In our one-year analysis encompassing all three SCs, we observed that the process times were generally consistent with those reported in multicenter trials IVT or ET [12–17]. Currently only 1% of all stroke patients are treated within the “golden hour”, which is also reflected in our results.

The American Stroke Association targets to achieve 85% of all thrombolyses within 60 min and ET within 90 min. The analysis shows that these targets can hardly be reached, particularly when using DNS or DND, and provides an incentive for further optimization of inhospital workflows. Currently, the median door-to-needle time is 41 min, with the 75th percentile at 61 min. The door-to-groin puncture time serves as a rough comparison, though not exact, and with a median of 108 min, it also exceeds the target times [18]. The results highlight the potential for improvements of intrahospital process times [5, 19, 20].

Notably, about 58% of the time from the initial alarm to imaging are apportioned to the pre-hospital phase, while 42% occurred within the hospital setting. Interestingly, patients with later confirmed strokes had 3.3 min faster pre-hospital alarm-to-door times and 10.2 min faster door-to-imaging times. Additionally, process times within the hospital were reduced to 34% for treated patients compared to 42% for untreated patients. These reductions can possibly be explained by enhanced vigilance among both, EMS personnel and neurologists in the EDs when dealing with stroke patients, where actual stroke or even eligibility for treatment can be suspected even before imaging. Nevertheless, the door-to-image-time should continue to undergo process optimization in order to reduce in-hospital delays and ensure the quickest possible imaging for the patient. Moreover, we observe a significantly faster onset-to-door time in treated compared to untreated patients, which, when considered together with the other pre-hospital process times, leads to the assumption that there is an awareness in the population—i.e., among the person who finds or alerts—that a corresponding therapy-needing severe symptomatology exists. However, further pre-hospital process times did not exhibit significant differences between treated and untreated patients, suggesting either efficient time management or reduced anticipation of treatment in the preclinical phase. Our comprehensive analysis of the entire rescue chain, spanning from the initial alarm to treatment, could uncover some potential to save time for all patients. Therefore, early confirmation of stroke suspicion and evaluation of treatment eligibility seems to be a promising model for a time-effective rescue chain. In addition, preponing neurological evaluation might improve the rate of confirmed stroke cases as among all suspected stroke transportations, which was now at 55% under the respective IVENA codes.

Analyses conducted in other cities showed similar challenges in the pre-hospital chain of care, which led to the establishment of MSUs, where a well-equipped stroke mobile including a mobile CT is used to allow for evaluation of IVT eligibility during pre-hospital care. In a recent analysis, the time saved with this strategy lead to an mRS reduction comparable to application of rt-PA in the 3–4.5-h time window [7]. At the moment, we see potential for improvement in the achieved median mRS of 3 in all treated patients, which we aim to address, among other things, by implementing time-saving measures, which should also improve the fraction of patients treated in the “golden hour”.

However, the immense costs for purchasing and operating MSUs raise uncertainties about cost-effectiveness [10]. Furthermore, and mainly due to cost constraints, MSUs have not been implemented as a standard practice for stroke care to date [7].

To circumvent these downsides of MSUs, we propose an expedited rescue chain with in-ambulance video-consulting. For this, EMS ambulances have to be equipped with telemedical hardware, such as a high-definition camera, mobile headsets, a managed mobile device, and a 5G router, allowing for two-way audio–video communication between EMS personnel and a transported patient in the ambulance with stroke experts at the admitting SC. Video-consultation should be performed during patient transportation and include the interprofessional communication between EMS personnel and the remote neurologist. Furthermore, guided NIHSS assessment could be conducted in-transit to the next SC to prove eligibility for IVT. By establishing the contact between patients and stroke experts as early as possible within the rescue chain, it is anticipated that processing times for confirmed stroke cases and especially patients with treatment eligibility can be significantly improved. Indeed, in-transit clinical assessment during transportation has been shown to significantly reduce door-to-needle times without compromising safety in previous trials [9, 21–23]. Alternatively, other concepts involving telemedicine have been able to significantly reduce the time to ET without introducing delays in IVT [24]. Both approaches appear to be advantageous and should contribute significantly to expedited care, potentially leading to improved outcomes. Especially the parallelization of elements of the rescue chain has high potential to reduce time losses in this scenario.

However, to our knowledge an effect of in-transit telemedicine on outcome in stroke patients has not been assessed yet. Furthermore, NIHSS assessment via telemedical devices has been shown to yield reliable results in the setting of MSUs [25, 26]. Hence, based on this evidence, implementation of such conception should be safe and effective and gives reason to expect advantageous for acute stroke care.

Additionally, our analysis pointed to an inferiority of processing times until groin puncture for ET patients in the setting of DnS compared to mothership or DnD concept. A large source of delay can be seen in the necessary organization of a secondary transportation to the CSC of DnS patients. Hence, relevant time saving can be expected, when CSC teams are involved in the telemedically supported emergency care early in these scenarios and the EMS teams stay with the patient until further transport requirements are clarified. Thereby, further delays for secondary transportation may be drastically reduced compared to the current system, where a second EMS team must be alarmed for secondary transportation to a CSC if the patient is eligible for ET.

As early imaging is the cornerstone for time-efficient treatment of stroke patients and IVT in telestroke networks has been widely used effectively and safe [21], we propose a hypothetical extension of the previously schematized rescue chain conception, which would comprise imaging and IVT at the next available scanner under telemedical in-transit supervision of a teleneurologist followed by direct transportation to the SC during thrombolysis in the ambulance car. This approach would combine the benefits of early imaging from MSUs, where CT is brought to the patient, and cost effectiveness by drawing on the broad availability of CT scanners in urban as well as even most rural areas in Western countries. Particularly patients in the city's outskirts may profit from this extension. According to the isochrone map in our study, 15 min could be saved in some parts of the city of Düsseldorf. Similar time savings can be expected for other cities and may be even enhanced for rural areas, where distances to SCs and therefore transportation times are even higher. A crucial point in the discussion of a new healthcare concept is the effectiveness and safety of the concept. The application of such telemedical in-transit models has already demonstrated their safety and effectiveness [11, 23]. While there are potential sources of errors in these models, such as connectivity issues, especially with large data transfers like video and audio, these issues have not proven to be significant. Moreover, in urban areas, there is widespread mobile network coverage, and problems with data transmission are less likely to occur. Other sources of errors, such as hardware or human errors, were rarely observed during implementation [23]. Nevertheless, prior training, especially regarding team communication, is crucial for ensuring smooth workflows [27].

However, this extended, patient centered approach is challenging regarding reimbursement, legal, and responsibility issues that must be clarified. Additionally, our isochrones analysis identified only a limited number of areas in the city that could benefit from such a hypothetical conception. Nevertheless, after safety evaluation in a pilot setting, rural areas might benefit to a greater extent. It has been shown that new concepts in stroke care, especially including telemedical approaches, could expedite the rescue chain and might improve outcome through time savings [26, 28].

To estimate the costs of our proposed concept, we commissioned a first health economic appraisal that compares the cost points of the concept with the current system. The economic scenario analysis shows that the novel concept could be potentially cost saving in regard to staff costs (see Supplementary Tab. 2). However, these cost savings for the medical staff have to be offset with costs for investment and operating charges of a telemedical system.

### Limitations

Our analysis has limitations. Firstly, our analysis could only include patients from our metropolitan region transported following IVENA notification. Consequently, our findings and plans for improvement must be reevaluated for other settings. Not all stroke cases were included, only those under the respective IVENA codes. Complete data were not available for a larger portion, but the recorded times of the individual intervals did not differ significantly between the complete and incomplete data.

## Conclusions

The utilization of in-transit telemedicine with the goal of minimizing delays in both the pre-hospital and in-hospital phases should be promoted in our setting. Prognostic analyses have suggested that expediting care within the rescue chain can be expected to yield improved long-term outcomes by facilitating faster treatment.

## Supplementary Information

Supplementary Material 1

## Data Availability

The datasets used and/or analysed during the current study are available from the corresponding author on reasonable request.

## Abbreviations

AIS: Acute ischemic stroke
CSC: Comprehensive Stroke Center
CT: Computed-Tomography
DnD: Drip-and-Drive
DnS: Drip-and-Ship
ED: Emergency department
EMS: Emergency medical services
ET: Endovascular treatment
HE: Health Economic
IVENA: Interdisciplinary Healthcare Proof System
ICH: Intracerebral Hemorrhage
IVT: Intravenous Thrombolysis
MRI: Magnetic Resonance Imaging
MSU: Mobile Stroke Units
mRS: Modified Rankin Scale
MSP: Mothership Principle
PMC: Primary Stroke Center
SC: Stroke Centers
SU: Stroke Unit
TIA: Transient Ischemic Attack
