# Supplementary material for: Prehospital telemedicine support for urban stroke care: Analysis of current state of care and conceptualization
Source: BMC Emerg Med. 2024 Nov 27;24:224. doi: 10.1186/s12873-024-01142-3 (PMC11600966; doi:10.1186/s12873-024-01142-3)
Supplement: Supplementary file 1 — Supplementary Material 1 [file 12873_2024_1142_MOESM1_ESM.docx]

Supplements

*HE assessment.*

Considering the total process costs, suspected stroke cases incur average staff costs of 131.91 € (median 112.05 €). Patients with stroke who received therapy have significantly lower staff costs (104.81 €) than the other groups (125.05 – 144.23 €) due to substantially lower average door-to-image times.

In our scenario analysis introducing in-transit telemedicine, we find that, on average, 38.93€ can be saved per patient (median 23.79€) in comparison to the status quo (Table A2). This corresponds to approximately 30% or, calculated with median times, 21% of total status quo costs for all suspected stroke cases. Particularly in the group of unconfirmed suspected stroke cases, which has the highest door-to-imaging times, staff costs of 46.67 € per patient could be saved. The use of in-transit-telemedicine is more favorable compared to the status quo as soon as the door-to-image time is at least reduced to an average of 40.50 minutes or calculated with median to 28.63 minutes.

There is already a cost neutrality between the concepts when the door-to-image-time is reduced from 43.86 minutes to 40.50 minutes on average if the transport times do not increase due to the in-transit-telemedicine. With the expected additional time savings in the door-to-image time frame, it is anticipated that staff cost savings of up to 38.93 € per patient could be achieved on average, through the optimization of treatment processes, a savings of up to 46.67 € per patient with unconfirmed stroke may be possible. Time and staff cost savings are expectable and in line with findings of other cost-analyses across Europe (9-11).

**Appendix A: Assumptions for the wage calculation for the professional groups**

*Calculation steps of gross wages per minute*

First, the monthly employer gross wages, as defined by the collective agreement, were multiplied by 12 to obtain the annual income, excluding any bonuses (except for a fixed 40 euros for emergency paramedics).

Next, the number of working days was determined, which amounted to 252 working days in 2022, after subtracting 30 vacation days.

The daily working hours were established at either 39 or 42 hours, depending on the specific group of individuals and the terms of the collective agreement. These daily hours were then divided by the 5 working days in a typical workweek.

Subsequently, the total working hours for the year were computed by multiplying the number of working days by the daily working hours, resulting in a figure of 39 (or 42) hours multiplied by 222.

Finally, the wage per minute was derived by dividing the annual income by the total working hours per year, and then further dividing by 60 (minutes).

Details for each professional group can be found in Table A1.

*Used times for analyses based on expert assumptions*

Paramedics and emergency paramedics are involved 100% of the prehospital time (action to leave-time from hospital).

Senior physicians are involved with a probability of 30% for 5 minutes in the door-to-imaging timeframe, e.g., in case of ambiguities; 30% for 2 minutes in image-to-needle timeframe, e.g., in case of ambiguities, and 8 minutes in the image-to-groin time frame (radiologist).

Assistant physicians are involved 100% (neurologist) or 5 minutes (radiologist) in the door-to-imaging timeframe; 100% (neurologist) or 8 minutes (radiologist) in the image-to-needle timeframe, and 80% (neurologist) and 15 minutes (radiologist) in the image-to-groin timeframe.

Emergency room nurses are involved 100% in the door-to-imaging timeframe and 80% in the image-to-groin timeframe.

Medical-technical radiology assistants are involved 10 minutes in the door-to-imaging timeframe and 15 minutes in the image-to-groin timeframe.

| **Professional group** | **Pay group** | **Experience level** | **Monthly gross wage in €** | **Working hours per week** | **Gross wage per minute in €** | **Collective Agreement for the Public Service - underlying agreement** |
| --- | --- | --- | --- | --- | --- | --- |
| Paramedic | E4/E5 (ratio 50:50) | 3 | 3631.68/3744.93 | 39 | 0.4259 | Area of the Federation of Municipal Employers' Associations |
| Emergency paramedic | P8 | 3 | 4243.86 + 40 | 39 | 0.4947 | Table for Care |
| Senior physician (university hospital) | Ä3 | 2 | 11013.61 | 42 | 1.1812 | Physicians at university hospitals |
| Senior physician (municipal hospital) | Ä3 | 2 | 10835.59 | 40 | 1.2202 | Physicians at municipal hospitals |
| Assisstant physician (university hospital) | Ä1 | 3 | 7106.21 | 42 | 0.7621 | Physicians at university hospitals |
| Assisstant physician (municipal hospital) | Ä1 | 3 | 6983.88 | 40 | 0.7864 | Physicians at municipal hospitals |
| Emergency department nurse | KR8 | 3 | 4349.22 | 39 | 0.5023 | Nursing staff in the public service of the federal states |
| Medical-technical radiology assisstant | E7 | 3 | 4117.54 | 39 | 0.4755 | Service of the Federal States |
| Pay group: Classification based on the professional; Experience level: Classification based on the years in the professional field | | | | | | |

**Table A1: Pay groups and wages per profession**

| **Savings in Euro** | **All** | **Confirmed stroke patients** | | **Unconfirmed stroke patients** | **Treated stroke patients** | **Untreated stroke patients** |
| --- | --- | --- | --- | --- | --- | --- |
| **Calculated with mean** | -38.93 | -33.40 | -46,67 | | -13.09 | -39.65 |
| **Calculated with median** | -23.79 | -19.96 | -28.89 | | -10.38 | -23.79 |

**Table A2: Savings (in €) of using new approach compared to old standard of stroke care**

Assumption of in-transit telemedicine 5 minutes and reduction of door-to-imaging time to 10 minutes
